# An Account of a White Swelling Successfully Treated

**Published:** 1781-03

**Authors:** 


### SECTION II.

### Essays and Observations.

I. *An Account of a White Swelling ſucceſsfully treated. By* F. Swediar, M. D. *Phyſician in London. Communicated by* Maxwell Garthſhore, M. D. F. R. S. *Read* March 19, 1781.

THE patient whoſe caſe I am going to relate was in perfect health, when in the month of November 1779, he was attacked in in the night with a flight fhivering, and the next morning found his knee fomewhat fwelled, but fo flightly, that it did not prevent his going out. At his return home he fat half an hour in his parlour, and then attempted to go up ftairs, but found himfelf unable to walk on account of the fwelling of his knee, which was now greatly increafed, with an acute pain he felt whenever he attempted to move the joint. He then went to bed; but the pain, inftead of diminifhing, increafed, and continued all night fo as to prevent his fleeping, obliging him to vary the pofition of the affected limb every four or five minutes. During all this time, however, he had not the leaft degree of fever.

The day following, by the advice of his phyficians, leeches, fomentations of white poppy heads, and faccharum faturni were applied to the knee, but without procuring the leaft relief. Electricity, and a variety of other remedies that are ufually prefcribed in cafes of this fort, were fucceffively had recourfe to for fifteen days; but during the whole of that fpace the pains continued with extreme violence, the patient was unable to move from his bed to the fire-fide without the affiftance of two per-

ſons, and the tumour, inſtead of leſſening, increaſed, and afforded evident marks of fluctuation within the capſular ligament. The real ſeat of the diſeaſe was at firſt exactly at the inferior part of the os femoris where the patella begins; and as the patient had experienced ſhiverings at times for two or three days, it was ſuppoſed to be a real ſuppuration, and poultices of bread and milk were applied in order to promote it. The firſt day they were uſed the pains were a little diminiſhed; but this relief was of ſhort duration, for they returned again the day following with the ſame violence as before.

The fluctuation within the joint being now evidently to be diſtinguiſhed by the feel, it was agreed to open the tumour. The opinions of the medical gentlemen aſſembled on this occaſion were different; ſome were for, others againſt the operation. While we were conſulting on this matter, I ventured to propoſe a remedy which I remembered to have ſeen tried five or ſix years before in a ſwelling of the knee, attended with a fluctuation as in the preſent caſe, but without any pain, except when the joint was moved. The firſt knowledge I had of this remedy, was from a friend who ſpoke of it as being very common among the peaſant peasants in Hungary. These people being frequently obliged to pass the night in the deserts and in the open air, are often troubled with this kind of tumour of the knee, with a similar fluctuation within the capsula exactly like a dropsy, which they soon cure by means of the remedy in question.

In the case I alluded to, and which occurred at Vienna, the patient had been visited by several of the most eminent physicians and surgeons of that city; but the complaint every day grew worse, so that at length the excessive pains the patient experienced whenever he attempted to walk, confined him to his room. In this state the Hungarian remedy was recommended. I was assured that in four days it usually cured. It was accordingly had recourse to, and with so much success, that at the end of four days the patient was sufficiently recovered to be able to walk out, and in eight days the cure was complete.

It was this same remedy I proposed to try in the present case, though the disease seemed different in its nature, symptoms, and progress. The other practitioners present readily consented to make the experiment, and in four days after the application of it, without any other external or or internal remedy, the patient experienced a considerable degree of relief; he began to ſleep in the night, and was eaſier in the day-time. At the end of five days the fluctuation entirely diſappeared; but there ſtill remained an enlargement of the inner condyle of the thigh bone, and the leg was much diminiſhed in ſize both above and below the tumour. The patient, likewiſe, now began to be troubled with violent pains in the region of the kidnies and back; but by perſevering in the uſe of the remedy, and by a liberal uſe of honey internally, he was in five weeks well enough recovered to be able to walk out, though lame. By the daily uſe of the fleſh-bruſh, however, the tumour of the condyle gradually diſappeared, the pains of the loins went perfectly off, and he has ſince enjoyed the moſt perfect health.

It ſeems right to obſerve, that the colour of the ſkin was never changed, either in this caſe or in the one I ſaw at Vienna.

The preſcription which proved ſo efficacious in theſe two inſtances is as follows:

℞. G. Ammoniac. ℥ij.

Aceti ſcillitic. q. ſ. ut fiat terendo linimentum, quod parti affectæ bis de die bene infricetur, ſuperponendo emplaſtr. ſeq. calefactum.

℞ G. Am-

℞. G. Ammoniac. ℥j.

Acet. fcill. q. f. ut fiat emplaftrum quo pars affecta tegatur.

Before the ointment is rubbed in, juniper berries fhould be thrown upon burning charcoal or a hot iron, and the vapour arifing therefrom well rubbed into the knee with a piece of flannel; after which the ointment may be ufed in the manner above directed.

N° 32, *Newman Street, Oxford Street.*

